# Supplementary material for: Non-conditioned bone marrow chimeric mouse generation using culture-based enrichment of hematopoietic stem and progenitor cells
Source: Nat Commun. 2021 Jun 11;12:3568. doi: 10.1038/s41467-021-23763-z (PMC8195984; doi:10.1038/s41467-021-23763-z)
Supplement: Supplementary file 1 — Supplementary Information [file 41467_2021_23763_MOESM1_ESM.pdf]

# SUPPLEMENTARY INFORMATION

Non-conditioned bone marrow chimeric mouse generation using culture-based enrichment of hematopoietic stem and progenitor cells

Ochi and Morita et al.

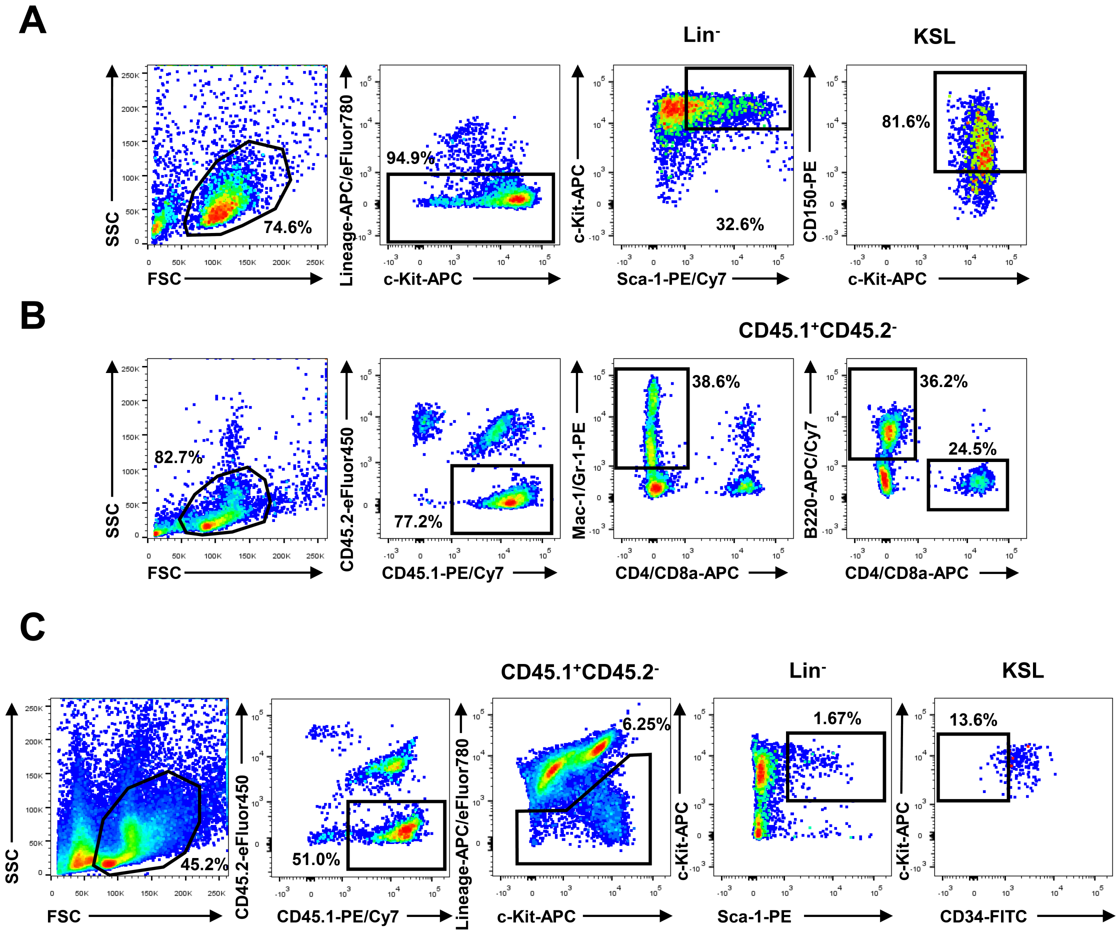


**Figure S1: Representative FACS gating**

(**A**) Representative gating strategy for the identification of $CD150^{+}KSL$ cells in cultures derived from c-$Kit^{+}$ BM cells.

(**B**) Representative gating strategy for the identification of myeloid cells (Mac-$1^{+}$/Gr-$1^{+}$), T cells $CD4^{+}$/$CD8a^{+}$), and B cells ($B220^{+}$) within the donor $CD45.1^{+}CD45.2^{-}$ population in PB samples.

(**C**) Representative gating strategy for the identification of $CD34^{-/lo}KSL$ ($CD34^{-}KSL$) cells among $CD45.1^{+}CD45.2^{-}$ population in BM samples.

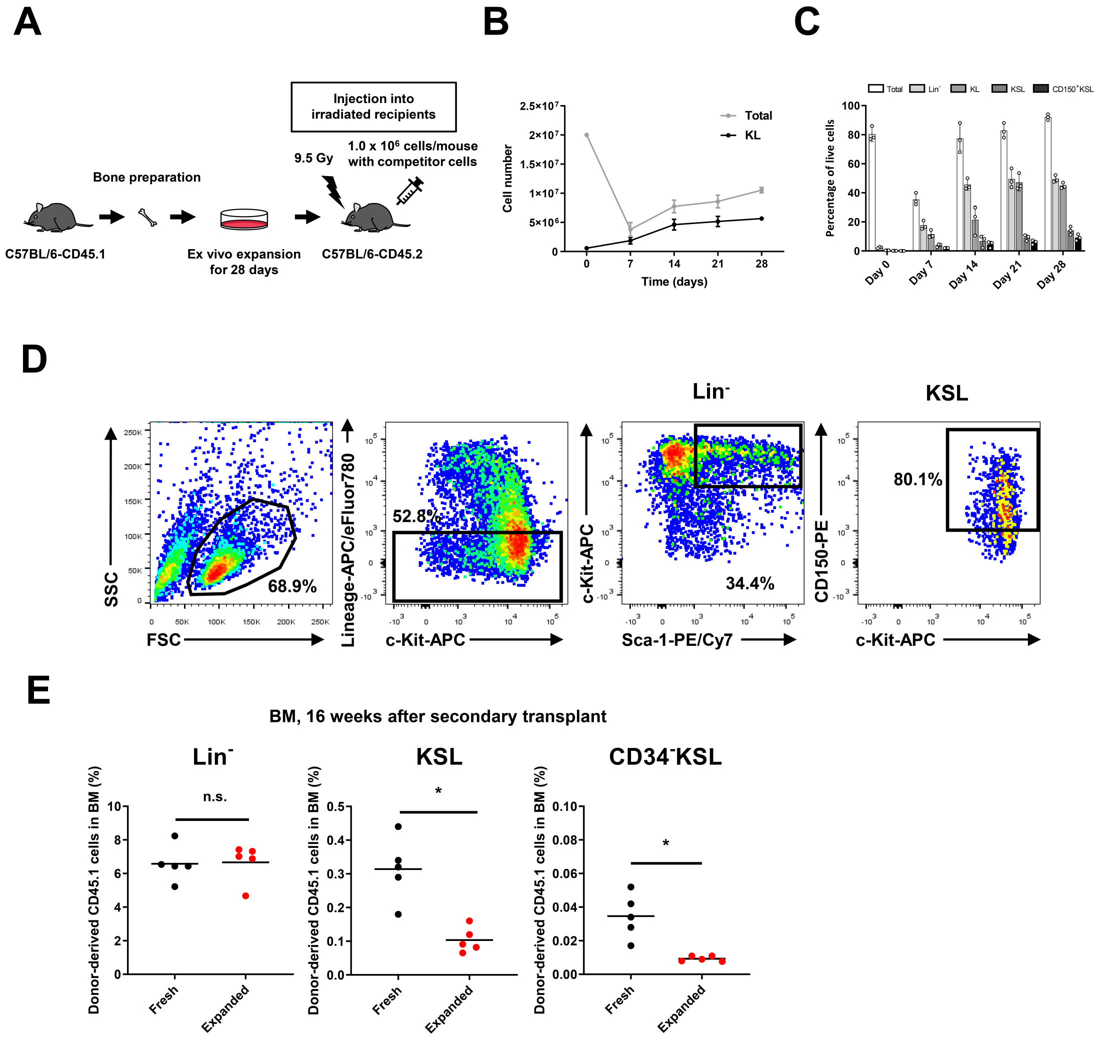


## Figure S2: Whole bone marrow cell cultures

**(A)** Experimental schematic: 2 x $10^7$ unfractionated whole BM cells were plated in PVA-based HSC media containing SCF and TPO, and cell cultures maintained for 28 days. Cell cultures were analyzed by cell counting and flow cytometry every 7 days. After 28 days, 1 x $10^6$ cultured cells were transplanted into 9.5 Gy irradiated C57BL/6-CD45.2 recipients (n = 5 mice per group) alongside 1 x $10^6$ C57BL/6-CD45.1/CD45.2 whole BM competitor cells, with donor PB chimerism quantified every 4 weeks for 16 weeks, at which point BM chimerism and secondary transplantation assays were performed.

**(B)** Total and KL cell numbers during the ex vivo culture. Mean ± SD of independent triplicate cultures.

**(C)** Percentage of Lin$^-$, KL, KSL, and CD150$^+$KSL phenotypic cell populations during the ex vivo culture. Mean ± SD of independent triplicate cultures.

(**D**) Representative gating strategy for the identification of $CD150^+KSL$ cells in cultures derived from whole BM cells.

(**E**) Percentage of $Lin^-$, KSL, and $CD34^-KSL$ cells in BM cells among donor-derived $CD45.1^+$ cells at 16 weeks in the secondary recipients described in Figure 1G. Mean ± SD from 5 primary recipients. Mean ± SD from 5 primary recipients (n = 5 mice per group). **p = 0.0016 in KSL, **p = 0.0030 in $CD34^-KSL$. Statistical significance was calculated using an unpaired two-tailed t-test, *p < 0.05, **p < 0.01; n.s., not significant.

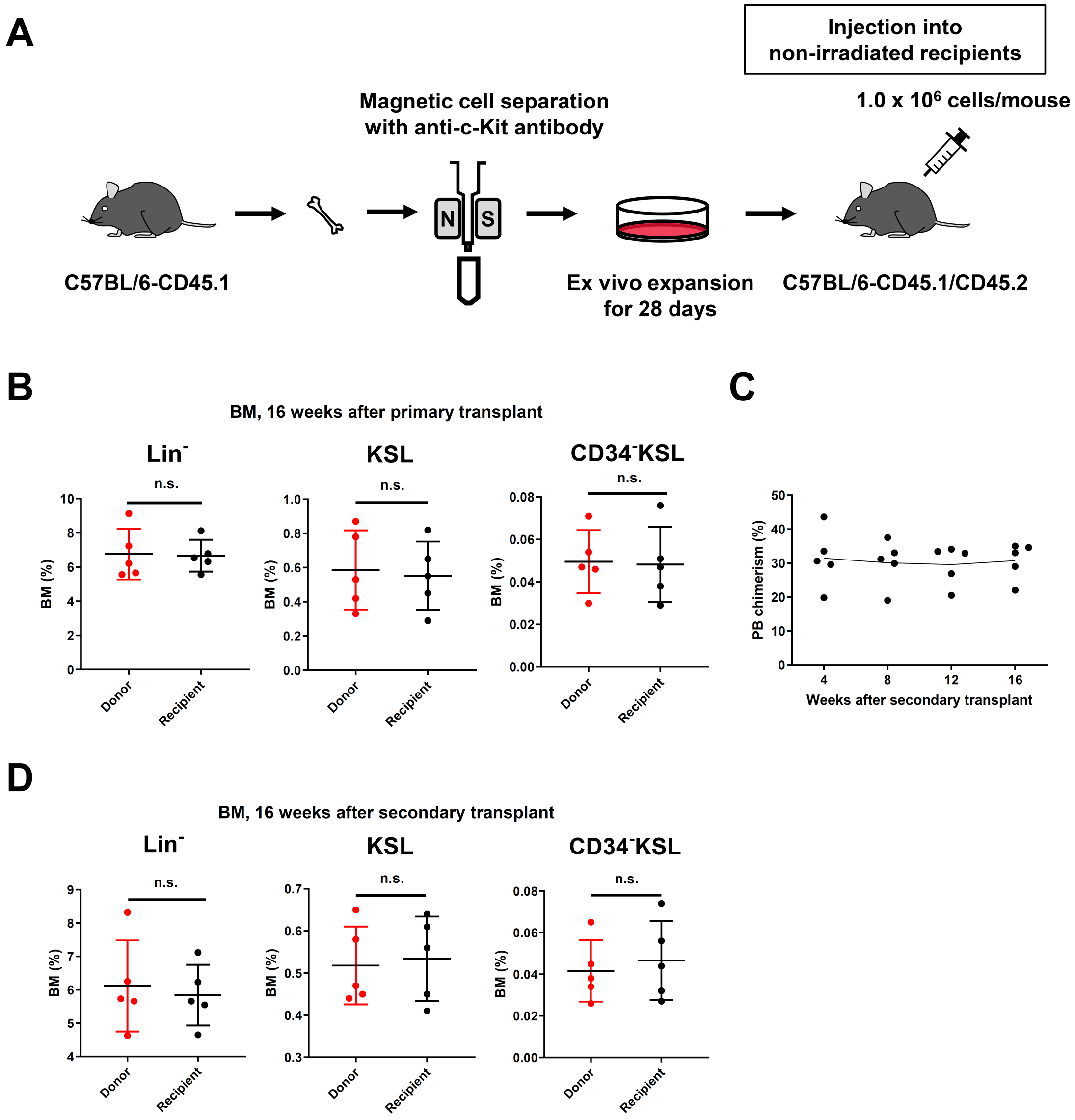


**Figure S3: Non-conditioned transplantation assays**

(A) Experimental schematic for the non-conditioned transplantation of culture-enriched HSPC cultures derived from c-Kit$^+$ BM cells.

(B) Percentage of Lin$^-$, KSL, and CD34$^-$KSL cells in BM cells among donor-derived CD45.1$^+$ or recipient-derived CD45.1$^+$CD45.2$^+$ cells at 16 weeks in non-conditioned primary recipients. Mean from 5 primary recipients (n = 5 mice per group). Statistical significance was calculated using an unpaired two-tailed t-test; n.s., not significant.

(C) Percentage of donor-derived CD45.1$^+$ cells in the PB of secondary recipients over time. (n = 5 mice)

(D) Percentage of $Lin^-$, KSL, and $CD34^-$KSL cells in BM cells among donor-derived $CD45.1^+$ or recipient-derived $CD45.1^+CD45.2^+$ cells at 16 weeks in secondary recipients. Mean from 5 primary recipients (n = 5 mice per group). Statistical significance was calculated using an unpaired two-tailed t-test; n.s., not significant.

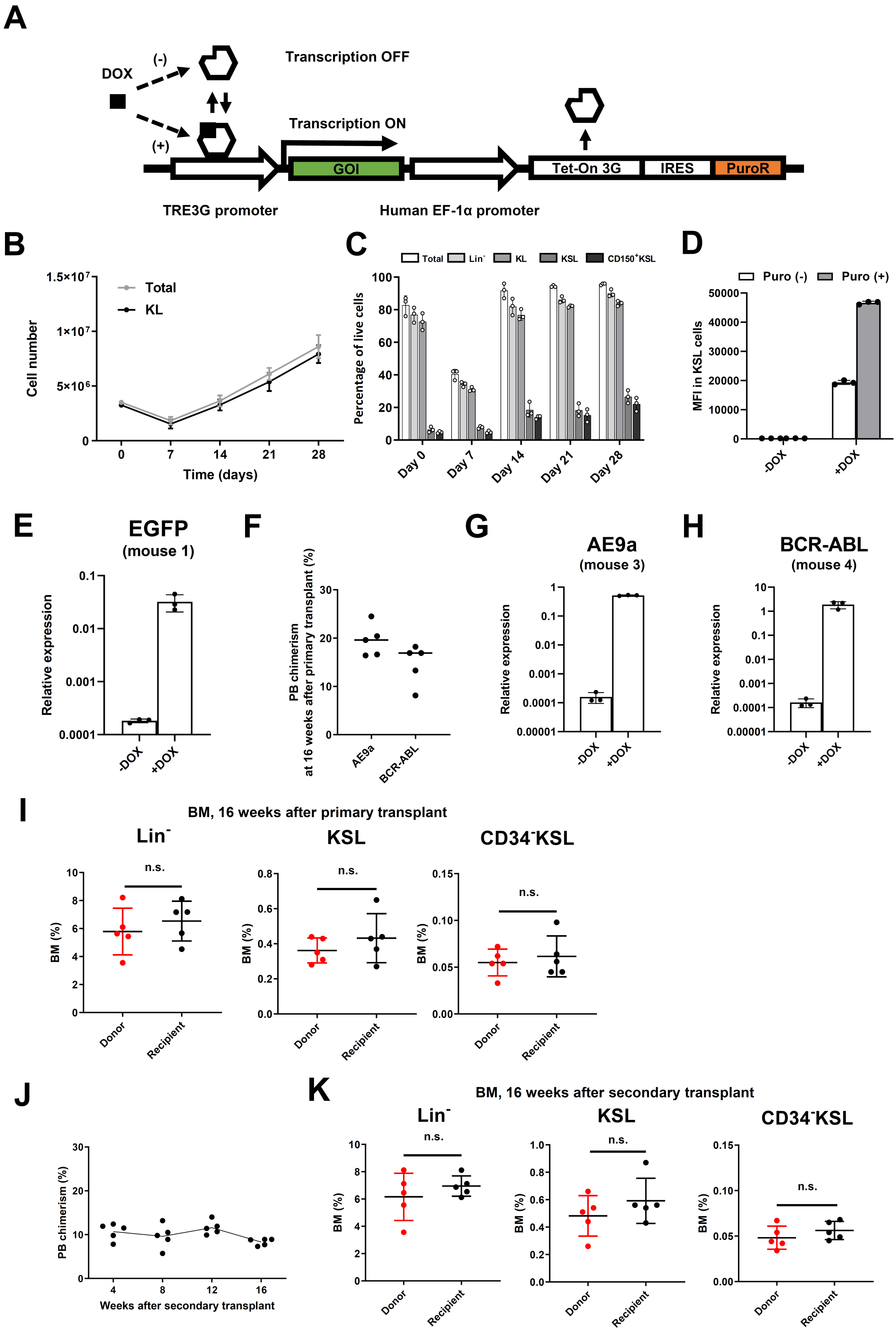
A
DOX
(-)
(+)
Transcription OFF
Transcription ON
GOI
Tet-On 3G
IRES
PuroR
TRE3G promoter
Human EF-1α promoter
B
Total
KL
Cell number
Time (days)
C
Total
KL
KSL
Percentage of live cells
Day 0
Day 7
Day 14
Day 21
Day 28
D
Puro (-)
Puro (+)
MFI in KSL cells
-DOX
+DOX
E
EGFP
(mouse 1)
Relative expression
F
PB chimerism
at 16 weeks after primary transplant (%)
AE9a
BCR-ABL
G
AE9a
(mouse 3)
H
BCR-ABL
(mouse 4)
I
BM, 16 weeks after primary transplant
KSL
n.s.
BM (%)
Donor
Recipient
J
PB chimerism (%)
Weeks after secondary transplant
K
BM, 16 weeks after secondary transplant

**Figure S4: Transduction of culture-enriched HSPCs**

(A) Schematic of the DOX-inducible EGFP expression vector including a puromycin selection marker. DOX: doxycycline, EF-1α promoter: elongation factor-1 alpha promoter, GOI: gene of interest, IRES: Internal ribosome entry sites, PuroR: puromycin-resistant gene.

(B) Total and KL cell numbers during the cell culture described in B. Mean ± SD of triplicate cultures.

(C) Mean percentage of total, $Lin^-$, KL, KSL, and $CD150^+$KSL cells. Mean ± SD of triplicate cultures.

(D) EGFP MFI in the KSL fraction of c-$Kit^+$ BM cultures with or without puromycin selection and with or without addition of DOX. Mean ± SD of independent triplicate cultures (n = 3)

(E) Relative gene expression of EGFP in $CD45.1^+$ PB cells before and after DOX administration (EGFP, mouse 1 as a representative). Mean of three independent experiments, with gene expression normalized to *Gapdh* expression. Error bar denote SD.

(F) PB chimerism following non-conditioned transplantation of AE9a and BCR-ABL transduced/selected and expanded c-$Kit^+$ BM cells at 16-week post-transplantation (n = 5 mice per group).

(G) Relative expression of AE9a in $CD45.1^+$ PB cells before and after DOX administration (AE9a, mouse 3 as a representative). Mean of three independent experiments, with gene expression normalized to *Gapdh* expression. Error bar denote SD.

(H) Relative expression of BCR-ABL in $CD45.1^+$ PB cells before and after DOX administration (BCR-ABL, mouse 4 as a representative). Mean of three independent experiments, with gene expression normalized to *Gapdh* expression. Error bar denote SD.

(I) Percentage of $Lin^-$, KSL, and $CD34^-$KSL cells in BM cells among donor-derived $CD45.1^+$ or recipient-derived $CD45.1^+CD45.2^+$ cells at 16 weeks in primary recipients following transplantation of EGFP-transduced and selected c-$Kit^+$ BM cells. Mean of 5 recipients (n = 5 mice per group).

(J) Percentage of donor-derived $CD45.1^+$ cells in secondary recipients over time, derived from EGFP-transduced and selected c-$Kit^+$ BM cells. (n = 5 mice)

(K) Percentage of $Lin^-$, KSL, and $CD34^-$KSL cells in BM cells among donor-derived $CD45.1^+$ or recipient-derived $CD45.1^+CD45.2^+$ cells at 16 weeks after secondary transplantation, derived from EGFP-transduced and selected c-$Kit^+$ BM cells. Mean of 5

recipients (n = 5 mice per group). Statistical significance was calculated using an unpaired two-tailed t-test; n.s., not significant.

**Supplementary Table 1: Antibodies used in this study**

| Antibody | Dilution | Source | Identifier |
|---|---|---|---|
| APC anti-c-Kit (2B8) | 1:100 | eBioscience | Cat# 17-1171-83 |
| PE anti-CD150 (SLAM) (TC15-12F12.2) | 1:350 | BioLegend | Cat# 115904 |
| FITC anti-CD34 (RAM34) | 1:100 | eBioscience | Cat# 11-0341-85 |
| PE/Cy5 anti-CD34 (MEC14.7) | 1:100 | BioLegend | Cat# 119312 |
| PE/Cy7 anti- Ly-6A/E (Sca-1)(D7) | 1:700 | eBioscience | Cat # 25-5981-82 |
| PE anti-Ly-6A/E (Sca-1) (D7) | 1:700 | BioLegend | Cat# 108108 |
| APC-eFluor 780 Ly-6G/Ly-6C (RB6-8C5) | 1:1400 | eBioscience | Cat# 47-5931-82 |
| APC-eFluor 780 CD11b (M1/70) | 1:1400 | eBioscience | Cat# 47-0112-82 |
| APC-eFluor 780 CD4 (RM4-5) | 1:1400 | eBioscience | Cat# 47-0042-82 |
| APC-eFluor 780 CD8a (53-6.7) | 1:700 | eBioscience | Cat# 47-0081-82 |
| APC-eFluor 780 CD45R (B220) | 1:700 | eBioscience | Cat# 47-0452-82 |
| APC-eFluor 780 CD127 (A7R34) | 1:350 | eBioscience | Cat# 47-1271-82 |
| APC-eFluor 780 TER-119 (TER-119) | 1:350 | eBioscience | Cat# 47-5921-82 |
| PE-Cy7 anti-CD45.1(A20) | 1:500 | Tonbo Biosciences | Cat# 60-0453-U025 |
| eFluor450 anti-CD45.2 (104) | 1:500 | eBioscience | Cat# 48-0454-82 |
| PE anti-Ly-6G/Ly-6C (RB6-8C5) | 1:2000 | eBioscience | Cat# 12-5931-82 |
| PE anti-CD11b (M1/70) | 1:2000 | eBioscience | Cat# 12-0112-82 |
| APC-eFluor780 anti-CD45R (RA3-6B2) | 1:1000 | eBioscience | Cat# 17-0452-83 |
| APC anti-CD4 (RM4-5) | 1:2000 | BioLegend | Cat# 100516 |
| APC anti-CD8 (53-6.7) | 1:2000 | eBioscience | Cat# 17-0081-83 |

**Supplementary Table 2: Primers used in this study**

| Primer name | Primer sequence |
|---|---|
| *p16$^{Ink4a}$* forward | GAACTCTTTCGGTCGTACCC |
| *p16$^{Ink4a}$* reverse | CGAATCTGCACCGTAGTTGA |
| *p19$^{Arf}$* forward | GGGTTTTCTTGGTGAAGTTCG |
| *p19$^{Arf}$* reverse | TTGCCCATCATCATCACCT |
| *Trp53* forward | CAGTCTACTTCCCGCCATAA |
| *Trp53* reverse | GTCTCAGCCCTGAAGTCATAAG |
| *EGFP* forward | AAGTTCATCTGCACCACCG |
| *EGFP* reverse | TCCTTGAAGAAGATGGTGCG |
| *AE9a* forward | CCACCTACCACAGAGCCATCA |
| *AE9a* reverse | AGCCTAGATTGCGTCTTCACATC |
| *BCR-ABL* forward | CCGCTGACCATCAATAAGGAA |
| *BCR-ABL* reverse | CTGAGGCTCAAAGTCAGATGCTACT |
| *Gapdh* forward | AACTTTGGCATTGTGGAAGG |
| *Gapdh* reverse | ACACATTGGGGGTAGGAACA |
