# Supplementary material for: Non-conditioned bone marrow chimeric mouse generation using culture-based enrichment of hematopoietic stem and progenitor cells
Source: Nat Commun. 2021 Jun 11;12:3568. doi: 10.1038/s41467-021-23763-z (PMC8195984; doi:10.1038/s41467-021-23763-z)
Supplement: Supplementary file 3 — Reporting Summary [file 41467_2021_23763_MOESM3_ESM.pdf]

nature research

Corresponding author(s): Satoshi Yamazaki

Last updated by author(s): Apr 21, 2021

Reporting Summary

Nature Research wishes to improve the reproducibility of the work that we publish. This form provides structure for consistency and transparency in reporting. For further information on Nature Research policies, see our Editorial Policies and the Editorial Policy Checklist.

Statistics

For all statistical analyses, confirm that the following items are present in the figure legend, table legend, main text, or Methods section.

n/a	Confirmed	
☐	☒	The exact sample size (*n*) for each experimental group/condition, given as a discrete number and unit of measurement
☐	☒	A statement on whether measurements were taken from distinct samples or whether the same sample was measured repeatedly
☐	☒	The statistical test(s) used AND whether they are one- or two-sided<br>*Only common tests should be described solely by name; describe more complex techniques in the Methods section.*
☒	☐	A description of all covariates tested
☒	☐	A description of any assumptions or corrections, such as tests of normality and adjustment for multiple comparisons
☐	☒	A full description of the statistical parameters including central tendency (e.g. means) or other basic estimates (e.g. regression coefficient) AND variation (e.g. standard deviation) or associated estimates of uncertainty (e.g. confidence intervals)
☐	☒	For null hypothesis testing, the test statistic (e.g. F, t, r) with confidence intervals, effect sizes, degrees of freedom and P value noted<br>*Give P values as exact values whenever suitable.*
☒	☐	For Bayesian analysis, information on the choice of priors and Markov chain Monte Carlo settings
☒	☐	For hierarchical and complex designs, identification of the appropriate level for tests and full reporting of outcomes
☒	☐	Estimates of effect sizes (e.g. Cohen's d, Pearson's r), indicating how they were calculated

Our web collection on statistics for biologists contains articles on many of the points above.

Software and code

Policy information about availability of computer code

Data collection	BD FACSuite
Data analysis	FFlowJo (v10.5.3) Software (FlowJo, LLC)<br>GraphPad Prism version 7 and 8 (GraphPad software)<br>Extreme Limiting Dilution Assay (ELDA) software (http://bioinf.wehi.edu.au/software/elda/)

For manuscripts utilizing custom algorithms or software that are central to the research but not yet described in published literature, software must be made available to editors and reviewers. We strongly encourage code deposition in a community repository (e.g. GitHub). See the Nature Research guidelines for submitting code & software for further information.

Data

Policy information about availability of data

All manuscripts must include a data availability statement. This statement should provide the following information, where applicable:

- Accession codes, unique identifiers, or web links for publicly available datasets
- A list of figures that have associated raw data
- A description of any restrictions on data availability

Data supporting the findings of this work are available within the paper and its Supplementary Information files. A reporting summary for this Article is available as a Supplementary Information file. The datasets and materials generated and analyzed during the current study are available from the corresponding author upon request. The source data underlying Figures 1, 2, 3, and 4, as well as Supplementary Figures 2, 3, and 4 are provided as a Source Data file. A detailed protocol is available at the Protocol Exchange.

Field-specific reporting

Please select the one below that is the best fit for your research. If you are not sure, read the appropriate sections before making your selection.

- [x] Life sciences
- [ ] Behavioural & social sciences
- [ ] Ecological, evolutionary & environmental sciences

For a reference copy of the document with all sections, see nature.com/documents/nr-reporting-summary-flat.pdf

Life sciences study design

All studies must disclose on these points even when the disclosure is negative.

Sample size	The sample size was determined based on the minimum number of animals/replicates required for the test calculations. The number of independent sample analyses is given in the figure legends for each experiment.
Data exclusions	No data were excluded.
Replication	For all figures, multiple independent experiments were performed and all attempts at replicating observation as described in the manuscript were successful.
Randomization	Recipient mice were randomly selected for transplantation groups.
Blinding	For data collected automatically by the instrument, the researcher was not blinded because observer bias was not expected to affect the results.

Reporting for specific materials, systems and methods

We require information from authors about some types of materials, experimental systems and methods used in many studies. Here, indicate whether each material, system or method listed is relevant to your study. If you are not sure if a list item applies to your research, read the appropriate section before selecting a response.

Materials & experimental systems

n/a	Involved in the study	
☐	☒	Antibodies
☒	☐	Eukaryotic cell lines
☒	☐	Palaeontology and archaeology
☐	☒	Animals and other organisms
☒	☐	Human research participants
☒	☐	Clinical data
☒	☐	Dual use research of concern

Methods

n/a	Involved in the study	
☒	☐	ChIP-seq
☐	☒	Flow cytometry
☒	☐	MRI-based neuroimaging

Antibodies

Antibodies used

APC anti-c-Kit (2B8) eBioscience Cat# 17-1171-83 (1:100)
PE anti-CD150 (SLAM) (TC15-12F12.2)BioLegend Cat# 115904 (1:350)
FITC anti-CD34 (RAM34) eBioscience Cat# 11-0341-85 (1:100)
PE/Cy5 anti-CD34 (MEC14.7) BioLegend Cat# 119312 (1:100)
PE/Cy7 anti- Ly-6A/E (Sca-1)(D7) eBioscience Cat # 25-5981-82 (1:700)
PE anti-Ly-6A/E (Sca-1) (D7) BioLegend Cat# 108108 (1:700)
APC-eFluor 780 anti-Ly-6G/Ly-6C (RB6-8C5) eBioscience Cat# 47-5931-82 (1:1400)
APC-eFluor 780 anti-CD11b (M1/70) eBioscience Cat# 47-0112-82 (1:1400)
APC-eFluor 780 anti-CD4 (RM4-5) eBioscience Cat# 47-0042-82 (1:1400)
APC-eFluor 780 anti-CD8a (53-6.7) eBioscience Cat# 47-0081-82 (1:700)
APC-eFluor 780 anti-CD45R (B220) (RA3-6B2) eBioscience Cat# 47-0452-82 (1:700)
APC-eFluor 780 anti-CD127 (A7R34) eBioscience Cat# 47-1271-82 (1:350)
APC-eFluor 780 anti-TER-119 (TER-119) eBioscience Cat# 47-5921-82 (1:350)
PE-Cy7 anti-CD45.1(A20) Tonbo Biosciences Cat# 60-0453-U025 (1:500)
eFluor450 anti-CD45.2 (104) eBioscience Cat# 48-0454-82 (1:500)
PE anti-Ly-6G/Ly-6C (RB6-8C5) eBioscience Cat# 12-5931-82 (1:2000)
PE anti-CD11b (M1/70) eBioscience Cat# 12-0112-82 (1:2000)
APC-eFluor780 anti-CD45R (RA3-6B2) eBioscience Cat# 17-0452-83 (1:1000)
APC anti-CD4 (RM4-5) BioLegend Cat# 100516 (1:2000)
APC anti-CD8 (53-6.7) eBioscience Cat# 17-0081-83 (1:2000)

Validation	All antibodies were validated by manufacturers for the applications and species used in this study. See manufacturers websites for validation statements (www.biolegend.com; www.thermofisher.com/us/en/home/life-science/antibodies/ebioscience ; www.bdbiosciences.com; www.cellsignal.com).<br>These antibodies are commonly used in our laboratory and have been published multiple times by us and other groups.

Animals and other organisms

Policy information about studies involving animals; ARRIVE guidelines recommended for reporting animal research

Laboratory animals	All mice were 8-12 weeks when experiments were started. Male and female mice were used in this study. The environment in the mouse chamber is a temperature of 23-25°C, humidity of about 50%, and a light period of 12 hours each.<br>C57BL/6 mice - The Jackson Laboratory (000664), Japan SLC, or Sankyo-Lab Service
Wild animals	This study did not involve wild animals
Field-collected samples	This study did not involve field-collected samples
Ethics oversight	All animal protocols were approved by the Animal Care and Use Committee of the Institute of Medical Science, the University of Tokyo

Note that full information on the approval of the study protocol must also be provided in the manuscript.

Flow Cytometry

Plots

Confirm that:

- [x] The axis labels state the marker and fluorochrome used (e.g. CD4-FITC).
- [x] The axis scales are clearly visible. Include numbers along axes only for bottom left plot of group (a 'group' is an analysis of identical markers).
- [x] All plots are contour plots with outliers or pseudocolor plots.
- [x] A numerical value for number of cells or percentage (with statistics) is provided.

Methodology

Sample preparation	Bone marrow, peripheral blood, and in vitro cell cultures were prepared into a single cell suspension in PBS. Bone marrow cells were cKit-enriched using an MACS LS column (Miltenyi). Red blood cell lysis using aqueous 140 mM ammonium chloride was performed on peripheral blood cells. Cells were filters (40uM) before FACS.
Instrument	BD FACS Verse
Software	BD FACSuite for data collection, FlowJo 10 for data analysis.
Cell population abundance	In this experiment, we did not use FACS for cell sorting.
Gating strategy	FFSC-A/SSC-A for mononuclear cells, FSC-H/FSC-W followed by SSC-H/SSC-W for singlets, PI/PE for PI- live cells. See Supplement Figure S1 for details.

- [x] Tick this box to confirm that a figure exemplifying the gating strategy is provided in the Supplementary Information.
